# Reducing traffic congestion in makkah during Hajj through the use of AI technology

**DOI:** 10.1016/j.heliyon.2023.e23304

**Published:** 2023-12-13

**Authors:** Foziah Gazzawe, Marwan Albahar

**Affiliations:** Department of Computer Science, Umm Al-Qura University, Mecca, Saudi Arabia

**Keywords:** AI technology, Digital transformation, Smart camera

## Abstract

This research addresses the pervasive issue of traffic congestion during the Hajj, where approximately 250,000 vehicles substantially exacerbate travel times and road accidents, while also escalating pollution levels, thereby adversely affecting public health. Aimed at bolstering the Kingdom's Vision 2030, the study focuses on the incorporation of artificial intelligence (AI) and advanced communication technologies to optimize traffic management in Mecca. Through the innovative deployment of smart cameras and real-time data analytics, the proposed system seeks to predict, manage, and alleviate traffic congestion by providing alternative routes and facilitating smoother vehicular movement. An exploration into the myriad benefits of this AI-integrated system reveals potentials such as enhanced road safety, improved emergency response efficiencies, and elevated air quality, thereby contributing to the overall wellbeing of the community and environment. In addition, the research anticipates that reducing traffic bottlenecks will indirectly invigorate local businesses and augment tourism revenues, aligning with the objectives of enhancing economic prosperity. By advocating a multi-faceted approach to crowd monitoring and management, this study underscores the indispensable role of AI in revolutionizing traffic management strategies, despite the challenges posed by the complexity of real-time data simulation and the unique intricacies of the Hajj.

## 1 Introduction

Hajj, the annual Islamic Pilgrimage, holds great significance for millions of Muslims who gather in the holy city of Makkah, Saudi Arabia. This momentous event takes place over a specific period each year and has been observed for 14 centuries. Generally, the Hajj pilgrimage has seen an increase in the number of attendees over the past years, up to the present, despite a slight decrease due to COVID-19. According to the [1]; the event attracted more than 140,000 internal attendees and over 700,000 from abroad. With an average of 3 million pilgrims visiting Makkah annually, the journey from Arafat to Muzdalifah has become a challenging task due to the increasing number of attendees [2].

The success and safety of mass gatherings, regardless of their nature or location, heavily rely on effective transport management. Among all the mass gatherings worldwide, the Hajj pilgrimage stands out as the largest annual event, attracting millions of pilgrims to Makkah. This pilgrimage involves a series of rituals at different sites, necessitating significant movements between them. Therefore, ensuring efficient transport management becomes crucial to maintain the smooth flow of the event.

During Hajj, pilgrims follow a specific sequence and timeframe for their movements. The journey commences at the Grand Mosque in Makkah, with most pilgrims proceeding to their designated camps in Mina for the first night. On the second day, pilgrims travel from Mina or Makkah to Arafat, where they spend the entire day on Arafat Mountain before reaching Muzdalifah before midnight. The third day involves pilgrims returning to Mina to perform the ritual of 'Stoning the devil’ at Aljamarat Bridge, followed by Tawaf and Sayee at the Grand Mosque in Makkah before returning to Mina. Finally, on the fourth and fifth days, pilgrims stone all three pillars at the Aljamarat Bridge, after which they may proceed to Makkah for the final Tawaf, thereby completing their Hajj journey.

One of the major challenges faced by Hajj authorities is managing and coordinating the movement of pilgrims. Among the various stages of the pilgrimage, transportation between Arafat and Muzdalifah, known as Al-Nafrah, presents significant logistical difficulties. In the past, pilgrims traveled these long journeys on foot or by camel, which was very tiresome [3]. As the event attracted more pilgrims, overcrowding became a challenge. Consequently, the Hejaz Railway was constructed, running from Damascus to Medina, to ease access to Makkah. Later, the Mashair Railway was built to connect the three main Hajj sites. Over the decades, this railway has expanded to accommodate 377,000 pilgrims [3]. Presently, transport systems have been enhanced with buses and modern trains. Despite several measures to enhance transport operations, such as vehicle scheduling and controlled access to train stations and sites, the sheer number of pilgrims continues to impact the efficiency of transport management. Challenges include limited road networks and inadequate bus capacity relative to demand, leading to congestion, traffic jams, longer travel times, and concerns about air quality. As a result, innovative solutions have been proposed to improve pilgrim transport between Hajj sites. Currently, transport is managed and organized by the Tawafa Establishments (TEs) in collaboration with the Hajj Transport Department and the Hajj Traffic Department. These establishments adhere to predetermined transport schedules and employ designated modes of transport to facilitate the movement of pilgrims between sites.

Efficient transport management necessitates meticulous planning to control the movements of both pedestrians and vehicles, prevent congestion, and mitigate any incidents that may arise. The Hajj Traffic Plan incorporates various measures to facilitate bus and collective transport vehicle movements in Makkah, including optimized pilgrim scheduling to alleviate congestion and overcrowding.

The Presidency of the Two Holy Mosques faces numerous challenges in managing the influx of visitors who come to perform Hajj or Umrah. Handling the movement of millions of visitors within specific geographic areas for varying durations presents a formidable task. Moreover, effective communication becomes an additional barrier due to the diverse languages spoken by the visitors, necessitating efficient guidance for religious observances. Additionally, visitor health is of paramount importance, and the Presidency is striving to provide high-quality services by embracing modern technology in line with Vision 2030.

Researchers have proposed various modern technologies to address the challenges encountered during Hajj and Umrah. Artificial Intelligence (AI) has emerged as a potential solution, with applications such as Image Processing and Computer Vision being utilized to leverage the extensive CCTV infrastructure in sacred places (Showail, 2022). AI has also played a significant role in enhancing teaching and education, providing a superior experience for visitors (Shambour & Gutub, 2021).

This research focuses on implementing AI technology, specifically by deploying smart cameras, to track and predict traffic congestion. The proposed system utilizes Machine Learning for image processing, incorporating a headcount algorithm for stampede detection. This algorithm periodically counts heads in each segment, followed by edge detection and Hough transformations for matching. It processes the images and stores them in a cache for comparison [4]. The system aims to facilitate the identification of alternative routes, such as converting entrance lanes into exit lanes, to improve traffic flow and mobility during peak times. Furthermore, it seeks to reduce traffic-related risks and accidents, thereby enhancing overall road safety. In emergencies, the system could enable first aid teams to respond more efficiently, potentially saving crucial time for individuals needing urgent medical attention. The proposed AI system, in the context of the Hajj pilgrimage, remains theoretical. No real-world implementation or testing has been carried out due to the complexities of managing moving crowds. CCTV surveillance, used in major cities like New York for traffic monitoring and management, suggests that similar technologies could be significant when applied in scenarios like the Hajj.

The primary contributions of this research are as follows:

1. The paper aims to address traffic congestion during the Hajj season in Makkah by harnessing the power of AI technology. Through the implementation of a smart camera system, traffic patterns can be tracked, congestion can be predicted, and effective management strategies can be implemented.
2. The research proposes the use of smart cameras to monitor traffic conditions in real-time. By analyzing traffic flow, the system can identify alternative routes and optimize traffic movement, such as converting entrance lines to exit lines, especially during peak periods.
3. The proposed system strives to minimize traffic-related risks and accidents by efficiently managing traffic flow and enhancing road safety during the Hajj season.
4. The research explores the advantages of integrating AI technology into a smart Mecca, which includes improved traffic control, reduced congestion, shorter travel times, and an enhanced overall experience for both visitors and service providers during the Hajj season.

## 2 Literature review

### 2.1 Current situation in Makkah traffic

According to Saudi Arabia's Vision 2030, it is anticipated that the Hajj population will increase to five million, as Makkah continues to expand [5]. However, the emergence of COVID-19 halted the Mecca event in 2020. In response, the Saudi government has been developing frameworks and arrangements to manage the large crowds. Part of this effort includes constructing three types of mobility infrastructure, connecting the new districts to the old city of Mecca, to ease crowding. These include tunnels for vehicles only, combined vehicle and pedestrian use, and pedestrian-only passages.

Due to overcrowding, pilgrims often experience stressful conditions and discomfort when trying to reach their destinations. Overcrowding can also lead to panic, crowd turbulence, rushes, and human and traffic bottlenecks. To address these challenges, the Saudi administration has implemented traffic plans to reduce congestion and enhance crowd safety measures. These plans involve constructing expansive corridors along roads, specifically designed to accommodate a massive flow of people. These corridors facilitate organized movement and minimize the risk of stampedes and accidents. They consist of broad bridges and multilayered routes that enable pilgrims to safely move across different levels, ensuring smooth pedestrian flow and significantly reducing road congestion. To minimize pedestrian-vehicle conflicts, specific roads have been designated for pedestrians and vehicles separately. Four roads have been allocated for pedestrian use, while five are designated for vehicles [6].

### 2.2 Benefits of crowd management

The establishment of an effective crowd control mechanism for large gatherings is essential due to the multiple risks associated with such events, which can lead to significant losses [7]. Managing large crowds is crucial for several reasons, including ensuring the safety and security of participants, maintaining order and smooth movement, promoting enhanced hygiene and health, being prepared for crises, and aiding in future planning [8].

Firstly, effective crowd management ensures the safety of all attendees, including visitors, event organizers, security personnel, venue operators, emergency services, management, and local authorities. It aims to prevent stampedes, accidents, and other hazards resulting from overcrowding by implementing safety measures such as detecting suspicious behaviors, queuing, screening participants, establishing emergency response measures, equipping security personnel, and minimizing high densities within the crowd [7,8]. These measures help minimize incidents, accidents, and loss of life, thereby ensuring the safety of all individuals involved.

Secondly, crowd management ensures orderly conduct during rituals at specific locations, facilitating organized participation without restrictions or chaos [9]. Thirdly, it plays a crucial role in enhancing hygiene and health by minimizing disease transmission risks among pilgrims through adequate sanitation, routine health checks, and medical facilities [7]. Fourthly, effective crowd management helps in minimizing losses and aids in future planning, allowing management to implement convenient mitigation measures based on past experiences. As a result, the overall experience of attendees is enhanced, encouraging more participation in such events [7].

Regarding technological approaches [10], proposed using the YOLO3 (You Only Look Once) technology for real-time detection and classification of pilgrims. This approach, however, was found to be time-consuming, less accurate, and ineffective in detecting small crowds. Similarly [11], employed the YOLO3 technology, but this method also had limitations, including being time-consuming and requiring substantial database space.

### 2.3 Smart camera and implementation

A smart camera is the integration of advanced image sensors and processors with the ability to perform image processing independently without the interference of human personnel [12]. Smart camera technology integrated with computer vision algorithms have the capability of estimating real-time crowd density, detecting anomalies, and analysing the pattern of movement [2]. After the images have been captured, the camera's processors perform high level image processing, including analysis of motion and face recognition, afterward the results of the compressed videos and images are transmitted via a network to centralized database or cloud for decision making by relevant authorities [12]. A smart camera consists of a video sensor, processing unit, and communication unit.

Smart camera technology has informed the management of crowd and traffic at various events. One common example is the 2022 Word Cup that was hosted by Qatar where over 20,000 smart cameras were installed across stadiums and public arenas. The cameras were fitted with facial recognition, heat mapping and crowd tracking to monitor flows, moods and prevent congestion [13].

### 2.4 Different types of smart cameras and their functions

**Fig. 1:**
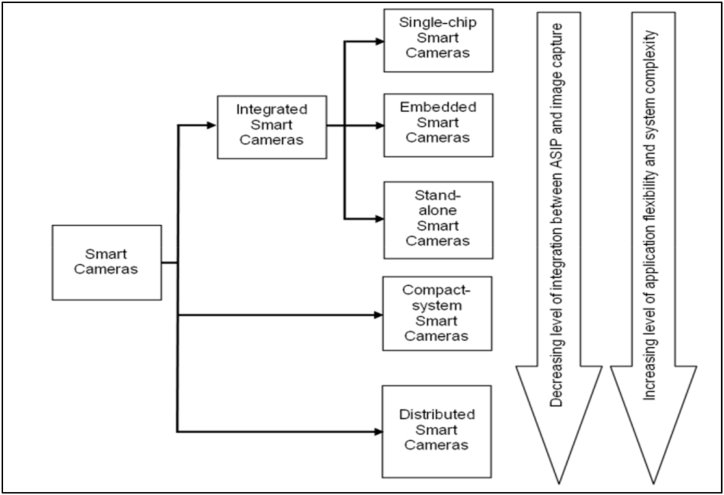
Classification of a smart camera based on integration levels [15].

Smart cameras can be classified based on the ASIP (Application Specific Information Processing) since it is the defining component [14]. This, therefore, classifies smart cameras into three groups, which include integrated smart cameras, distributed smart cameras, and compact system smart cameras. The integrated smart cameras can be further subdivided into smart cameras on a chip, embedded smart cameras, and stand-alone smart cameras. Fig. 1 shows a summary of the classification together with the integration level between the image capture parts and the ASIP. Additionally, it shows the level of flexibility of the smart cameras, which means the degree to which they can be reprogrammed or adapted to various kinds of applications [14].

a. Single-chip smart camera

In a single-chip camera, all components of its ASIP are done on the same chip as a CMOS image sensor. This sensor contains intelligent image processing circuits and an image sensor array parallel on the same chip, hence yielding smart sensors. Also, it is advantageous in terms of low power consumption, efficiency, very limited form factor, and small device count. However, single-chip cameras have pitfalls which include less flexibility due to large hard-wired functionality, high cost of manufacturing, and less modular design [14].

b. Embedded smart cameras

An embedded smart camera refers to a camera inside a device such as the camera on smartphones [14]. The camera may also be hidden in a device where it cannot be noticed by people. Example of embedded smart cameras include vision based optical mice, cameras on robots, vision-based fingerprint readers and cameras embedded in automobile applications. In these cameras the 10.13039/100011306ASIP functionality is supported by either device's processor or a dedicated processor [14].

c. Stand-alone smart cameras

A stand-alone smart camera resembles normal cameras such as general-purpose industry cameras or CCTV cameras. In this camera, the 10.13039/100011306ASIP functionality is supported by dedicated embedded processors that are run by intelligent algorithms [14].

d. Compact system smart cameras.

A compact camera is a normal camera connected to a separate, external, and dedicated image processor nearby using communication interfaces or dedicated cables [14]. The camera captures images and may carry out some ASIP functionality. For instance, it may perform pre-processing to minimize data amount or extraction of features. This type of camera is advantageous in terms of less expensive and easily upgraded or replaced. Also, the external processor has more memory which enables more processing power.

e. Distributed smart cameras.

Distributed smart cameras are jointly networked to carry out a similar task, this means that the data processing is performed jointly for a desired ASIP functionality [14]. The distributed framework presents a powerful and novel computing platform that avoids various touch problems associated with single smart camera. These cameras are widely applied in video surveillance, automobile, and machine vision. As much as these cameras have advantages, there exist disadvantages which include calibration challenges and coordination among the cameras on the network [14].

## 3 Problem statement

Crowded events, including the Hajj pilgrimage, pose significant challenges in communication and management, leading to potential risks and disruptions. The sheer number of participants increases the likelihood of stampedes and medical emergencies, demanding urgent and coordinated disaster control measures [4]. The 2015 stampede incident in Mina is among the deadliest incidents to be recorded in history. Over 2000 pilgrims lost their lives while thousands of others left injured [16]. This incident shows how overcrowding can lead to fatalities and tragedies if not managed effectively. Moreover, the diverse origins of attendees from various countries result in transportation difficulties, especially when navigating unfamiliar routes back to their destinations. To tackle these issues, there is an immediate need for a comprehensive disaster control and management system that leverages AI technology to enhance communication and traffic management during crowded events like the Hajj. Such a system must facilitate seamless communication between event organizers, security personnel, medical teams, and participants to enable rapid response and coordinated action during emergencies. Additionally, it should integrate intelligent traffic management functionalities to reduce congestion and ensure smooth participant movement throughout the event.

This research aims to develop and implement an AI-driven disaster control and management system as a robust solution tailored to the unique challenges of managing crowded events. The proposed system seeks to improve safety, minimize the risk of stampedes and medical crises, and optimize transportation logistics for participants with diverse backgrounds. The successful implementation of this research will ultimately contribute to a safer, more efficient, and well-organized experience for all attendees during the Hajj pilgrimage and other crowded events. It is critical to address how Artificial Intelligence would revolutionize communication and transportation during times of crisis. One key component of this system is its ability to seamlessly integrate with existing communication networks and infrastructure. Leveraging AI algorithms for real-time data analysis emergency services, social media platforms and sensors, the system is capable of accurately assessing potential risks. Thereafter, it would timely and effectively communicate relevant stakeholders. Additionally, the machine learning component employs machine learning algorithms that continuously emulate from past occurrences and accordingly maximize the response strategies.

## 4 Methodology

This research adopted a comprehensive approach by utilizing both primary and secondary data sources. The primary focus was on gathering firsthand observations from similar events to gain insights into the crowd management challenges faced during crowded gatherings like the Hajj pilgrimage. Additionally, secondary data from previous studies was collected to supplement and validate the findings obtained from firsthand observations. As recommended by Saraiva et al. (2018), the secondary data was subjected to repeated readings to ensure a thorough understanding and identification of potential issues through secondary research. This iterative process allowed the researchers to delve deeper into the data and uncover critical patterns and trends related to crowd management during large-scale events. A significant portion of the secondary data was derived from an extensive review of literature on the management of crowds during the Hajj in Saudi Arabia. This literature review included academic papers, reports, government publications, and relevant conference proceedings. By scrutinizing this literature, the researchers aimed to discern the strategies and practices adopted by the Saudi government in effectively managing the massive influx of pilgrims during the Hajj pilgrimage.

The information gathered from previous studies encompassed various aspects, such as the current technologies employed in crowd and traffic management, existing frameworks for handling large crowds, and the challenges encountered during past Hajj events. This data played a crucial role in shaping the objectives and direction of the proposed study. Through a rigorous assessment of the weaknesses and observations from the collected data, the researchers identified potential areas for improvement in crowd management during crowded events. These insights formed the basis for the proposed study, which focuses on the integration of smart cameras in crowd management. The proposed study aims to leverage smart camera technology to enhance crowd management efficiency and mitigate risks associated with overcrowding during the Hajj pilgrimage. By building upon the knowledge derived from the secondary data sources, the researchers seek to design and implement a sophisticated smart camera-based system that will facilitate smoother traffic flow, reduce congestion, and enhance overall safety for all attendees during the Hajj and other crowded events.

The deployment of smart cameras to manage crowds during the Hajj pilgrimage raises serious ethical considerations that should not be disregarded. One critical factor to consider is the possible invasion of privacy by smart cameras. These gadgets can take comprehensive photographs and videos of people in crowds, raising worries about data privacy and individual rights. Another ethical issue is consent and informed involvement as it may be difficult to acquire complete permissions from persons who may be captured by cameras in a crowded situation such as the Hajj pilgrimage. Ethical approval address how the privacy of participants would be protected throughout the research, ensuring that their names and personal information were not violated. This issue has been addressed by providing posters with information regarding the availability of CCTV surveillance within the premise.

### 4.1 Research design

The study adopted a qualitative research approach to investigate crowd management strategies during the Hajj pilgrimage in Mecca. The choice of a qualitative approach was made to gain an in-depth and comprehensive understanding of the complex social phenomena surrounding the crowd situation during the Hajj event. Qualitative research is well-suited for exploring intricate and multifaceted social phenomena, making it an ideal fit for studying crowd management during the Hajj, given its complexity.

This approach allowed the researchers to delve deeply into the subject matter, uncovering nuanced insights and perspectives from various stakeholders involved in crowd management. By employing qualitative methods, the study sought to capture the perceptions, practices, and experiences of all parties engaged in crowd management, including event organizers, security personnel, medical teams, and the participants themselves. These diverse perspectives provide a holistic view of the crowd management strategies and shed light on the challenges faced by different stakeholders in handling the massive crowds during the Hajj. In-depth interviews, focus group discussions, and direct observations were used as primary data collection methods to gather rich and contextual information from participants.

These qualitative techniques enabled the researchers to explore the intricacies of crowd management in real-life situations and understand the decision-making processes and strategies employed by various stakeholders. The qualitative findings of this study provide valuable insights into the effectiveness of existing crowd management strategies during the Hajj pilgrimage. By comprehensively understanding the dynamics of crowd management in Mecca, the research contributes to the development of more informed and evidence-based approaches to handle crowds during large-scale events.

The choice to use qualitative research methods alone to explore complex social phenomena such as crowd management during the Hajj pilgrimage is not by chance; it is based on several critical reasons. One reason is that qualitative research provides for a more in-depth examination and comprehension of crowd experiences, viewpoints, and social interactions (Zuhriddin Juraev, Ahn, and Hyun, 2023). Quantitative data may be difficult to gather and interpret in this setting because of the magnitude, unpredictability, and access limitations during the Hajj activities. By applying qualitative approaches such as observation and in-depth interviews, researchers are able to discover valuable information that illustrates the complexities of crowd behavior, decision-making processes, and the collective dynamics in action [17].

### 4.2 Research participants

This study employed purposeful sampling to select key stakeholders as participants, ensuring a comprehensive exploration of crowd management strategies during the Hajj pilgrimage in Mecca. The selected stakeholders represented a diverse group, including security personnel, religious authorities, event management officials, pilgrims, ICT experts, medical personnel, and other crowd management experts. Upon communicating with the relevant authorities, only those who gave their consent and fall within the criteria were selected. Security personnel and event management officials were recruited directly through relevant government departments overseeing Hajj operations. Religious authorities were approached through affiliations with the Ministry of Hajj and Umrah. Medical personnel were identified through major hospitals in Mecca and Mina. ICT experts were selected based on involvement in smart city technologies relevant to Hajj. Pilgrims were volunteers recruited through Hajj travel groups.

Purposeful sampling was chosen as the sampling technique because it allowed the researchers to intentionally select participants who possessed specific expertise and experiences relevant to crowd management during the Hajj. By including representatives from different stakeholder groups, the study aimed to capture a wide range of perspectives and insights into the complexities of crowd management during the event. The use of purposeful sampling facilitated a targeted and purpose-driven recruitment process, ensuring that the participants were knowledgeable and directly involved in crowd management efforts. Each participant's expertise and experiences were considered, enabling the researchers to gather in-depth information from different angles and areas of specialization.

By involving security personnel, religious authorities, event management officials, pilgrims, ICT experts, medical personnel, and other crowd management experts as participants, the study benefited from a wealth of diverse perspectives. These varied viewpoints provided a comprehensive understanding of the challenges, successes, and potential improvements in crowd management during the Hajj pilgrimage. Through interviews, focus group discussions, and observations, the researchers were able to gather valuable insights from each participant group, further enriching the study's findings. The collective knowledge and experiences of the key stakeholders contributed to a robust analysis of crowd management strategies, leading to more informed and nuanced recommendations for enhancing the overall safety and organization of the Hajj pilgrimage.

### 4.3 Data collection techniques

This study employed a mixed-methods approach, utilizing multiple data collection techniques to gain a comprehensive understanding of crowd management strategies during the Hajj pilgrimage in Mecca. The combination of data collection techniques included semi-structured interviews, observation, and analysis of data from secondary sources such as pictorials, videos, and published information from relevant sources. Semi-structured interviews were conducted with key stakeholders, including security personnel, religious authorities, event management officials, pilgrims, ICT experts, medical personnel, and other crowd management experts. During the semi-structured interviews, participants were asked a wide range of key questions to gather information and gain insights. Some of the specific aspects covered included their experiences with the subject matter, their thoughts on the following topics: the use of AI for crowd management, the effectiveness of smart cameras, and ethical concerns about using smart cameras, as well as any challenges or successes they may have encountered in relation to these topics. Participants offered a variety of perspectives on the use of AI for crowd management, with some emphasizing the potential benefits and others expressing doubts. The idea that AI has the ability to dramatically improve crowd management by offering real-time data analysis and prediction skills emerged as a recurring discovery from these discussions. This might help authorities in developing more effective crowd-control and crowd-direction methods during events or emergencies, eventually contributing to greater safety measures [18]. One key area of inquiry was the participants' own experiences and viewpoints on this topic under consideration. This enables a more in-depth assessment of their ideas, feelings, and attitudes regarding the employment of AI to regulate crowds These interviews were instrumental in eliciting valuable knowledge, perspectives, and experiences related to crowd management during the Hajj pilgrimage. The open-ended nature of semi-structured interviews allowed participants to share in-depth insights and provide nuanced information on their roles and observations during the event. Observation played a crucial role in the data collection process. Pictures and crowd management technologies were carefully observed to gain a clear overview of the crowd situation during the Hajj. By observing visual data, the researchers could gain valuable insights into crowd dynamics, congestion points, and the effectiveness of existing crowd management technologies in practice.

Furthermore, data from secondary sources, such as videos, were collected and closely analyzed to examine the correlation between crowd behaviors during the Hajj and potential strategies for crowd control and management. These videos were collected from multiple sources including Aerial footage from Saudi news media helicopters covering the Jamarat stoning rituals in 2020 and 2022, Photos and videos publicly shared by pilgrims on social media during the 2016–2022 Hajj, and compilations of footage from multiple Hajj seasons by news outlets like the BBC, CNN and Al Jazeera. The video analysis provided supplementary information to support the findings derived from interviews and observations, enriching the research with real-life scenarios and examples. To strengthen the validity and credibility of the research, data from certified publications and articles on the subject matter and domain were incorporated as secondary sources. These published materials contributed to a more comprehensive literature review and provided additional context and insights into crowd management strategies during the Hajj pilgrimage.

### 4.4 Data analysis

The data analysis process for this study involved a thematic analysis of the transcripts from interviews and notes from the observations. Thematic analysis is a rigorous and iterative approach that allowed the researchers to uncover meaningful patterns and insights related to crowd management during the Hajj pilgrimage. The first step in thematic analysis was to assess and familiarize with the data gathered from interviews and observations. This enabled the researchers to acquire a thorough knowledge of the participants' opinions as well as the observed crowd. NVIVO 12 qualitative data analysis software was used to evaluate the interview transcripts and observational notes. NVivo's visualization tools like hierarchy charts and comparison diagrams were used to identify main codes containing meaningful information pertaining to crowd management techniques, crowd composition and characteristics, bottlenecks, best crowd evacuation practices, and the technologies used during the Hajj pilgrimage.

These codes served as initial indicators of important themes within the data. Subsequently, the identified codes were organized into themes based on similarities and patterns. NVivo facilitated efficient data management, coding, and theme identification from a sizable qualitative dataset management practice during the Hajj event. The researchers looked for recurring concepts and patterns across the data to group related codes under common themes. This process allowed for a systematic organization of the data, making it easier to identify overarching patterns in crowd management strategies and practices.

Several approaches were used to establish intercoder reliability and validity of the analysis since many researchers were involved in the coding process to assure the reliability and accuracy of the identified themes. Intercoder reliability metrics such as Cohen's kappa were calculated to determine coders' consistency. Furthermore, the researchers refined the themes through collaborative analysis sessions by critically reviewing and establishing consensus on the relationships between codes and their relevance to their research objectives. These rigorous processes for ensuring intercoder agreement and validity guaranteed that thematic analysis was carried out consistently and objectively across the research team. This iterative process of theme refinement helped strengthen the validity of the findings and ensured that all significant aspects of crowd management during the Hajj were thoroughly explored. Data management and organizing were initially difficult due to the large amount of qualitative data obtained through interviews and visual data. The research team overcame this by meticulously organizing and coding the enormous transcripts and notes with NVIVO software.

The pilgrims who participated in the interviews recorded a positive experience regarding their visit to the holy site. They highlighted some drawbacks and registered an improvement on the way crowd management practices have been improving from time to time. The ICT personnel noted an advancement in technology over the years, accidents and stampedes have been minimized and efforts to upgrade the technologies have progressively been applied.

## 5 Proposed model

The use of Robotics and AI-enabled IoTs has been widely accepted to carry out duties that are complex to human workforce. The proposed model will use advanced technology by integrating smart ICT infrastructure along a smart city. The infrastructure includes robots, smart cameras, communication devices and other IoT technologies within the domain. The force model will be used within this infrastructure pathway to control and manage traffic.

Audio devices such as speakers and coloured warning lights are initiated to pass across information about the stampede situation and how to move in the lanes. Using the RFID tags as identification tools for the affected people, the system would access their personal and health information and appropriate action taken. For example, some individuals might have underlying health conditions that may cause them to trip, and others may fall due to their old age [4]. The bracelet is used to store personal data and its design as Internet of Things can monitor pilgrims’ health conditions such as heart rate, temperature, blood oxygen. Additionally, according to Ref. [19] the bracelet has a functionality which enables pilgrims request for emergency services and security assistance during an emergency.

**Fig. 2:**
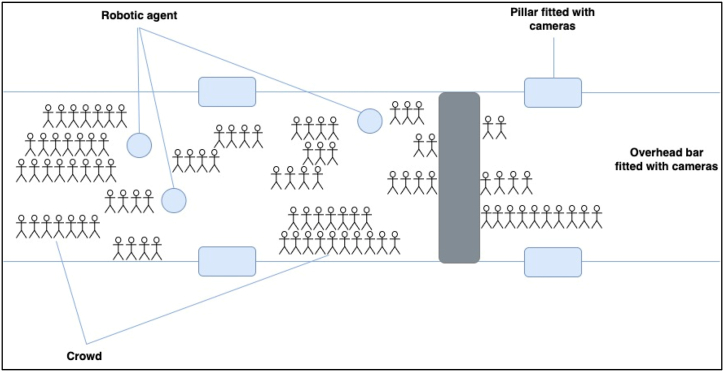
Model representation of the movement and AI technology use.

Thereafter, drones and robots would be used to make deliveries in terms of medical personnel and medication and rescue team to manage the situation [19]. Rescuing the individuals would require a standby ambulance at a strategic location to offer first-aid and transfer individuals to medical facilities for further medical intervention. Fig. 2 is a model representation of the movement of pilgrims and the location of AI technology along the path. Actor's symbol represents the crowd, blue circles represent the robotic agents roaming among the crowd, the rectangular bocks on the sides represent pillars fitted with monitoring and warning cameras along the path, while the overhead bar covers a wider view and has cameras fitted in all angles. Cameras are also embedded on drones which hover overhead and along the sides. These cameras have coloured lights for warning the crowds and are AI enabled to monitor the behaviour of the pedestrians as they move along the path.

### 5.1 The force model

Pilgrims and cars represent agents who possess various parameters such as speed, size, time, and position required for the stone throwing. The continuous force model is therefore applicable since the path followed by the pilgrims and cars is a continuous 2D space. In this space, agents move freely with three types of forces namely:

a. Internal forces

These forces make the agent move towards the target. The motivation within an individual is what drives him/her towards a specified destination or target.

b. External forces

These are repulsive forces originating from other agents using the same path. For instance, individual i may come across individual j who has suspicious motives or looks dangerous. This would instil fear in individual i, forcing him/her to move away on increase pace.

c. External forces originating from the path walls

These are attractive forces that may originate from friends, artists or audio communication that may result to grouping among the crowd.

The sum of forces affecting the movement of agents along the path is denoted by vector F

(1) $$\vec{F_{i}}=\vec{F_{{\text{des}}_{i}}}+\sum_{j\neq j}\vec{F_{\text{ij}}}+\vec{F_{\text{wi}}}$$

Fdes denotes the internal force of the agent.

(2) $$\vec{F_{\text{des}}}=m\text{.}\vec{a_{\text{des}}}$$

F_ij_ denotes the force originating from the centre of one agent to another agents, Bi to Bj

(3) $$\vec{F_{\text{ij}}}=\left\{ \begin{array}{l} \left(\frac{\vec{F_{j}}\cdot \vec{b_{\text{ij}}}}{\vec{b_{\text{ij}}}\cdot \vec{b_{\text{ij}}}}\right)\vec{b_{\text{ij}}},\text{if}\left|\vec{b_{\text{ij}}}\right|<r\\ 0,\text{otherwise} \end{array}\right.$$

b_ij_ = direction vector from Pj to Pi

(4) $$\vec{b_{\text{ij}}}=\left(x_{i}-x_{i}\right)i+\left(y_{i}-y_{i}\right)j$$

F_wi_ denotes the force originating from the walls of the path.

(5) $$\vec{F_{w_{i}}}=\left\{ \begin{array}{l} \left(\frac{k_{w}}{d}\right)\vec{W_{1}},\text{if}\;d>r\\ 0,\text{otherwise} \end{array}\right.$$

where:

K = constant for appropriate range of force

d = distance perpendicularly from the wall to the agent

r = agent's influence range.

Wi = shortest unit vector to the first agent Pi from the wall.

Agent parameters are updated periodically, ie at every simulation time step to determine the new location (L) at a time (t)

(6) $$\vec{L_{1}\left(t\right)}=\vec{L_{1}}\left(t-\text{Ts}\right)+\vec{V_{1}}\left(t-\text{Ts}\right)*\text{Ts}+\frac{1}{2}*\vec{a_{1}}\left(t-\text{Ts}\right)*\text{Ts}*\text{Ts}$$

V(t) = velocity at a time t

(7) $$\vec{V_{1}\left(t\right)}=\vec{V_{1}}\left(t-\text{Ts}\right)+\vec{a_{1}}\left(t-\text{Ts}\right)*\text{Ts}$$

a_i_ is the acceleration of the moving agent Pi at a time t, calculated as:

(8) $$\vec{a_{1}}\left(t\right)=\frac{\vec{F_{i}}\left(t\right)}{m_{i}}$$

### 5.2 An example simulation of the force model

In this example we assume that each agent has a desired velocity towards the destination. We have 10 individuals within the path at different positions, with desired velocities set towards the destination. For each time step, the simulation steps are as follows:

Step #1 Calculation of forces which are internal forces, external forces and forces from obstacles i.e from the path walls.

Sum up the three forces to get the resultant force acting on the agent (Internal force + External force + Obstacle force)

Step #2 Update the Velocity

The velocity of each agent is updated based on the mass and acceleration of the agent. Using Newton's second law of motion, acceleration is computed as.

F = ma.

Where F = force; m = mass; a = acceleration.

Step #3 Position Update

The position of each agent is updated based on the new velocity (Velocity *time step)

Step #4 Repeat Steps 1, 2 and 3 for the desired number of timesteps until the simulation is complete.

### 5.3 Advantages of using the force model in crowd management during the Hajj

i. Safety

This model provides a procedural comprehension and management of forces within a crowd. Therefore, the analysis of the forces acting on the agents enables authorities to identify areas of risks where there is congestion, accidents, and stampedes. Consequently, they appropriate measures are undertaken towards the safety of the pilgrims.

ii. Efficient resource allocation

Authorities allocate resources strategically when they have a clear understanding of forces within a crowd hence ensuring a smooth flow of the crowd. Examples of such resources to appropriate regions include barriers, alternative routes, medical personnel, signage, and security.

iii. Enhanced decision-making

This model, using the mathematical representation of forces within the agents enables authorities to assess the situation and offers tailored interventions and possible control measures. As a result, they can use the most appropriate control strategies to enhance safety, control the behaviour of the crowd and optimization of the experience of the pilgrims.

iv. Risk mitigation and assessment

The force model enables the authorities to assess and identify potential vulnerabilities using factors such as crowd movement speed, density and external factors influencing their movements.

v. Predictive analysis

Using the force model, crowd data can be used to predict future behaviours hence enabling them to have mitigative measures on critical situations for safety of the pilgrims.

In this study, we will not carry out the real practical simulation using the force model, rather we will adopt the level of service standard developed in the work of [20]. We use six levels of agent flow rate i.e from level A to F with corresponding parameters as shown in Table 1 below.

**Table 1:** The six level flow rates of pedestrians (Alazbah and zafar, 2019).

Level of Service	Density (ped/m^2^)	Space (m/ped)	Flow Rate (ped/min/m)	Av. Speed (m/s)
A	<0.27	>3.24	<23	>1.3
B	0.43 to 0.31	2.32 to 3.24	23 to 33	1.27
C	0.72 to 0.43	1.39 to 2.32	33 to 49	1.22
D	1.08 to 0.72	0.93 to 1.39	49 to 66	1.14
E	2.17 to 1.08	0.46 to 0.93	66 to 82	0.76
F	>2.17	<0.46	>82	<0.76

The flow states are further categorized into four states: A to C for free flow hence pedestrians walk at normal speed; D for constant flow hence pedestrians are required to adhere to the flow speed; E for crowded flow hence pedestrians are required to walk slowly due to limited walking space; and F for stampede state where pedestrians lose balance hence a disruption in movement [20].

**Fig. 3:**
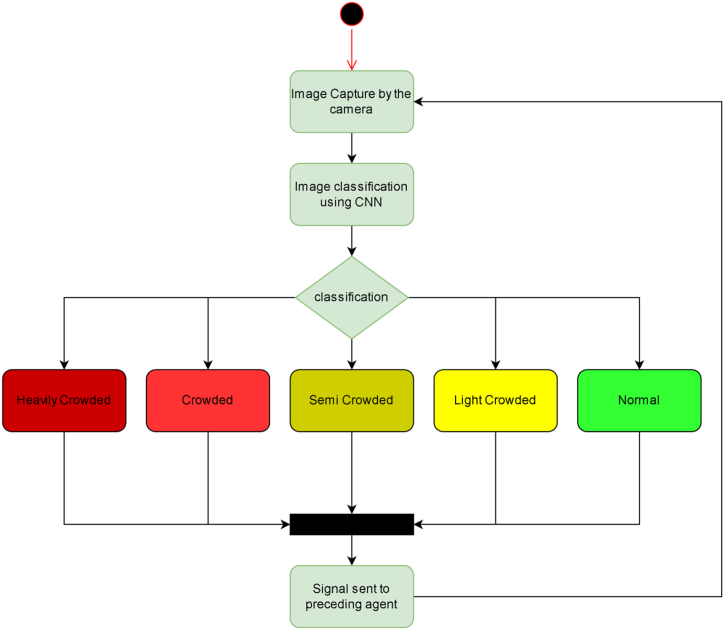
Model algorithm of the coloured warning camera lights.

Fig. 3 above presents a model state diagram showing how an image is captured by a camera, further classified into five different coloured lights using CNN and a signal sent to the preceding agent to communicate on the state of congestion. Cameras are fitted along the path, on pillars on the sides, overhead bars and drones. Also, AI-enabled robots are fitted with camera, sensors, and audio devices to roam around the crowd [19]. The overhead cameras take images of the moving crowd periodically and the image of the crowd is fed into a CNN algorithm for classification [20]. The crowd density is classified into five categories i.e heavy crowd, crowded, semi-crowd, light crowd or normal [21]. This classification of crowd is denoted by five different colours; heavy crowd is denoted by Dark Red, Crowded (Light Red), Semi-Crowd (Yellow), Light Crowd (Light Yellow) and Normal (Green). Whenever there is a crowded area along the path, the cameras trigger warning lights to the previous region before the crowd [20]. The meaning of each light colour is communicated to the crowd in advance through the MIS during the arrival. For instance, dark red light means that crowds should stop moving as they are approaching a heavy crowded region; Light-red light indicates a crowded area, therefore the crowd should move slowly when approaching the region; the crowd should decrease their moving pace when heading to semi-crowded region, denoted by yellow; light-yellow also requires slow movement; while green light indicates safety of the situation therefore normal movement [21].

### 5.4 Advantages of using smart camera to manage traffic

1) A smart camera possesses ability to capture, compute and cache data.
2) Support time sensitive applications by reducing latency and traffic on the links and increases response speed.
3) A smart camera can filter and process data before it is sent into the cloud, hence reducing traffic on the network and the overhead processing of the data on the cloud.
4) There is possibility of a smart camera performing access restrictions on the data before it is transmitted to the cloud, thus enhancing security for objects with less processing power and memory.
5) The nodes in the smart camera spread intensely in covering areas of intense crowding, as in the case of Hajj [14,22].

### 5.5 Limitations of using the force model

1) Inability to fully simplify real-world scenarios: The force model only relies on forces like friction, applied forces and gravity. Crowd such as that of the Hajj is affected by several variables like personal intentions, cultural practices, emotions and dynamics in social interactions. Therefore, a simple force model cannot fully integrate these variables effectively.
2) Inability to accurately quantify the forces: It is difficult to measure and accurately quantify forces within a dynamic crowd due to crowd's characteristics such as social behavior, emotions and the unpredictive nature of the crowd.
3) Conflict with ethical and cultural sensitivities of the crowd: The Hajj is a religious event where there is involvement of different cultures and ethical considerations, therefore the force model may fail to honour the pilgrims' cultural practices and beliefs.
4) Dynamics in crowd behaviour: Differences in priorities, response to stimuli and motivation in individuals may hinder the effectiveness of the forces model. This is due to the assumption of the model that the characteristics and response to forces of individual s within the crowd are uniform.
5) Effect of unforeseen events: External factors such as weather changes, security incidences and emergencies may not be accounted for by the force model. These factors significantly influence the crowd dynamics

In the event the crowd is not managed properly in the highly crowded region a stampede might occur. This could happen if the management or relevant personnel does not monitor the behaviour of the coloured lights. According to Ref. [23]; stampedes are the main cause of accidents during any crowd gathering. Whenever there is a stampede or an emergency and is communicated to the AI system to draw frames and identify the affected region, crowd control barriers should come hand in hand as a containment measure [21]. Mobile barriers should be preferred in barricading the affected frame/region in the crowd path for the purpose of minimizing further collisions as necessary action is taken by the management. Diversion is another management strategy that aims to direct the remaining crowd towards alternative lanes and routes to the desired destination (Shao, Shao and Kuo, 2019).

Therefore, as soon as the crowds detect an emergency, the authority should communicate and direct them to follow alternative routes or switch to other lanes. However, stampedes are not the only challenges but also violent behaviours and terrorism [24]. Consequently, there is need to put up security measures to detect weapons and violent behaviours in the crowd. As soon as they are detected by the system using the smart camera, swift action should be taken without instilling panic [24]. For instance, when such are detected, the individuals should be identified and isolated before harm is caused.

The use of mind maps is essential in crowd movement and management of the anomalies [25]. The information and infrastructural designed should be communicated earlier to the crowds so that they are aware of certain situations. For instance, manuals with pictorial illustrations should be distributed to all the pilgrims for easier comprehension. Also, during the induction and convergence, the authority should use messaging and screens fitted displaying videos of similar situations and actions expected to be taken in management of the same. The crowd should be aware of the road markings which guides on the direction and variations in the paths such as narrowing and diversions (Shao, Shao and Kuo, 2019). The use of traffic signals for communication is crucial in this type of crowd management; LED lights communicate danger by flickering red light while safety using green light [26]. Communication and signages should be in both English and Arabic languages. English is an international language, hence integrating it with Arabic language would be convenient for those pilgrims and other stakeholders who are not conversant with Arabic.

Additionally, the signals could include road signs showing the texture and gradient of the path to enable the crowd to adjust their movement patterns in terms of speed or diversions to relevant lanes. It would help the aged or individuals with health conditions make decisions on which lanes to use and which lanes to avoid and whether they need assistance at particular points. Apart from the age and health conditions, there exist other crowd dynamics that should be considered. This includes the direction of crowd movement, crowd size and density, gender, and emotions [27]. Such dynamics would influence the movement patterns causing turbulence and stops. Smart cameras fitted in strategic points can detect the dynamics and trigger emergency response [28]. For instance, the camera using AI can detect sudden stops and rapid turbulence which would indicate aged, children or individuals with health conditions who need attention.

## 6 Results and discussion

Various crowd management strategies have been revealed by the study, these strategies would be effective in ensuring that there is safety, effective evacuation mechanisms, minimization of stampedes and a smooth movement of the pilgrims during the Hajj. For instance, strategies such as infrastructure planning, effective communication mechanisms, crowd flow management, integration of AI technology and collaboration among various stakeholders have been of great significance. Similar application of this strategy has been used by the Shanghai Disneyland in China. Upon its opening, the premise recorded many attendees in history. As a result, there was need to establish an efficient crowd management infrastructure to handle the huge crowd, giving the attendees a positive experience and seamless movement. The crowd management infrastructure involved the establishment of an advanced transport network which included a dedicated metro line linking the park with the nearby airports and the downtown of Shanghai [29].

Varied perspectives and insights from various key stakeholders taking part in crowd management bring out effective strategies, bottlenecks and areas of improvement. As a result, there is provisions for more effective and holistic strategies towards crowd management during the Hajj pilgrimage. Also, the lessons learned from past crowd management experiences form the basis for future improvements for similar cases thus giving room for early detection and control of the crowd whenever there are emergencies.

**Fig. 4:**
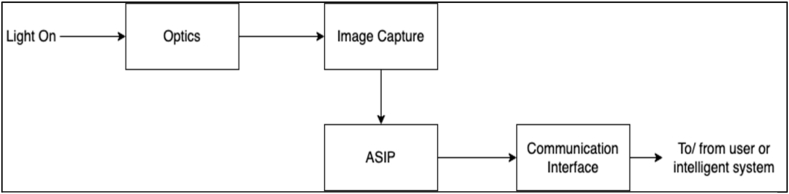
Workflow structure of a typical smart camera.

**Fig. 5:**
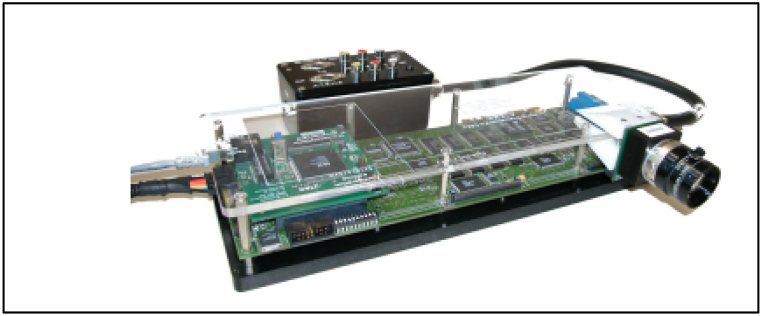
Architectural design of a smart camera prototype.

The smart camera uses the image capturing functionality to capture the crowd and vehicles in the way of matching. Normally, for a camera to capture an image, it must be illuminated by light which uses the optics [4]. The image is captured and processed through the ASIP functionality. The information is shared through a dedicated network, which is the communication interface of the camera system. The personnel monitor the behaviour of the AI data to make informed decisions. Fig. 4 below shows the typical functioning of a smart camera while Fig. 5 present the architectural design of a smart camera prototype.

There is limited knowledge on how incidences of confusion among the crowd are captured. It is therefore suggested that the real data in the real domain be trained in a knowledge-based system so that the implementation is more practical. The previous study does not illustrate how AI technology is used to manage crowds during Hajj. It only talks of the tunnels and fails to illustrate the technology used to assign the crowd and vehicles to their respective routes.

### 6.1 Integration of various technologies for crowd management during stampede

**Fig. 6:**
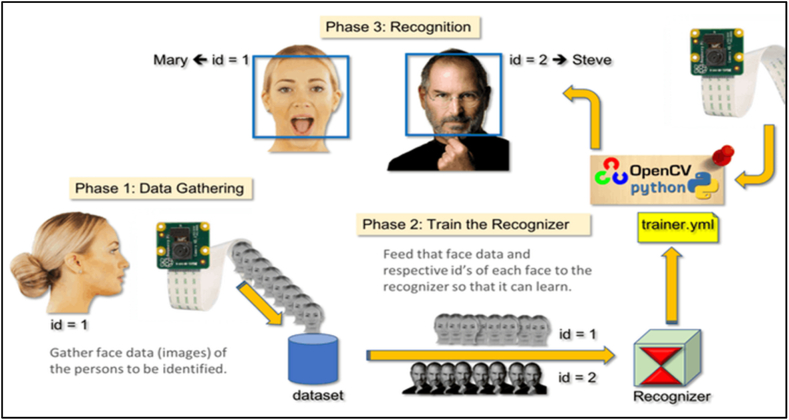
Face detection and recognition using OpenCV [32].

This illustration is aimed at solving the challenges associated with stampede in crowds, such problems include injuries and deaths of individuals [30]. We will use various technologies including, smart cameras, RFID, Machine Learning (ML), Artificial intelligence (AI) and smart city embedded devices. Smart cameras are integrated with RFIDS, AI and ML capabilities and are linked to a central database and intelligence system that is monitored by the city management personnel. The people who attend the Hajj need to register first and be given RFID tags upon arrival. The RFID functionality is used to read the RFID tags on the pilgrims [31], by focussing on a targeted segment of the crowd, the camera would capture the images of the pilgrims at the focus area and process the data through face recognition using machine learning and OpenCV [32]. Fig. 6 below illustrates the algorithm of face detection and recognition using OpenCV.

Surveillance and capturing of the crowd motion is done real-time to determine the variations in patterns of movement. Whenever an abnormally is detected in the motion and population of the moving crowd, the camera will send the information to a knowledge-based system where relevant personnel are standby for immediate action. For instance, a sudden reduction in headcount and an abrupt change in motion pattern would indicate a stampede (S. K [33].

Therefore, the system would initiate commands to the smart city infrastructure such as using Infrared (IR) to draw frames among the crowd using different colours with different meanings. Red colour is used to indicate red zones where entry should be denied or restricted. By marking the border-frame in which the stampede happens, the lighting would restrain the other crowd in motion to either stop or change the movement lanes. Table 2 below illustrates the possibility of a stampede using frames due to the abrupt decrease in the number of heads between the neighbouring segments. In frame 1, the head count changed from 120 to 100, hence there is a possible stampede. Whereas frame 2 maintained the headcount, hence normal movement of the crowd.

**Table 2:** Possibility of a stampede due to the abrupt decrease in heads.

FRAME 1	FRAME2
120 heads	132 heads
100 heads	132 heads

Audio devices such as speakers and coloured warning lights would be initiated to pass across information about the stampede situation and how to move in the lanes. Using the RFID tags as identification tools for the affected people, the system would access their personal and health information and appropriate action taken. For example, some individuals might have underlying health conditions that may cause them to trip, and others may fall due to their old age [4]. The bracelet is used to store personal data and its design as Internet of Things can monitor pilgrims’ health conditions such as heart rate, temperature, blood oxygen. Additionally, according to Ref. [19] the bracelet has a functionality which enables pilgrims request for emergency services and security assistance during an emergency.

## 7 Limitations of the research

This research has several limitations that need to be acknowledged. Firstly, the study exclusively focuses on the use of smart cameras at fixed positions along the path for crowd monitoring and management. As a result, it does not explore the potential benefits or challenges of using mobile cameras, such as those embedded in drones, for real-time monitoring or crowd warnings. Further research is required to investigate the feasibility and effectiveness of employing mobile camera technologies in crowd management during the Hajj pilgrimage. Secondly, the study primarily emphasizes pedestrians as the main agents along the path and does not consider other crowd compositions, such as those involving vehicles or mixed crowds of pedestrians and vehicles. The exclusion of vehicular crowds limits the generalizability of the findings to real-life scenarios where traffic congestion and interactions between different types of crowds may significantly impact crowd management dynamics. Future research should strive to encompass a broader range of crowd compositions for a more comprehensive understanding of crowd management strategies during the Hajj.

Moreover, this research heavily relies on data sourced from other scholars' theoretical work and does not engage in the actual simulation of the proposed smart camera model. The absence of real-time data simulation may affect the accuracy and practical applicability of the proposed solution. Data collected from static agents give different results as compared to moving agents. To validate the effectiveness of the proposed smart camera model for crowd management, future research should involve real-time simulations using computer vision and artificial intelligence algorithms. For instance, researchers could use simulation software to recreate crowded scenes during the Hajj and assess the ability of smart camera networks to accurately analyze and monitor crowd movement, behavior, and density. Using Machine Learning algorithms, models learn from past experiences and data for more enhanced decision-making. As a result, stakeholders could adopt the technology which offer tailored solutions that meet the challenges incurred during the pilgrimage.

In conclusion, these limitations underscore the need for further investigation and expansion of the current research scope. Future studies should explore the use of mobile cameras, consider diverse crowd compositions, and incorporate real-time data simulations to enhance the reliability and robustness of the proposed crowd management model. By addressing these limitations, researchers can advance the understanding and implementation of effective crowd management strategies during the Hajj pilgrimage and other crowded events.

## 8 Conclusions

This research has discussed the functionality of a smart camera that is either an embedded or a distributed system. For instance, the smart camera involves image capture using image sensors, ASIP functionality for data processing and a network for information communication [12]. The processing involves video analysis algorithms in transforming a network camera into a smart camera. The smart camera network enables real-time tracking of crowd sizes, densities, and flows. This provides the pilgrims and authorities with critical data to proactively identify high-risk areas and take preventive measures. Since smart cameras use AI to automatically analyze crowds, reliance on error-prone human estimation is reduced and faster response time is achieved. Smart camera's capability of communicating the state of the crowd and warning the pedestrians before entering the crowded region is more effective especially when the pilgrims are aware of the meaning of different colours of warning lights. As a result, stampedes are minimized, hence a decrease in casualties or injuries. In addition to improving safety, smart cameras' improved crowd management and coordination may improve pilgrims' entire experience by minimizing congestion, confusion and delays. Pilgrims may better focus on their spiritual endeavours with smoother crowd movements and real-time coaching. For the smart camera to function effectively in crowd management during Hajj, the city should be converted into a smart city with state-of-the AI infrastructure and framework. We propose that real data be captured during the Hajj, upon which training would be performed so that the crowd management is informed by real situations for the effectiveness of AI algorithms.

## Funding statement

This research has received no fund.

## Data availability statement

Datasets used to support the findings of this study are included within the article.

## CRediT authorship contribution statement

**Foziah Gazzawe:** Writing - review & editing, Writing - original draft, Visualization, Validation, Software, Resources, Methodology, Investigation, Formal analysis, Data curation, Conceptualization. **Marwan Albahar:** Writing - review & editing, Writing - original draft, Supervision, Funding acquisition.

## Declaration of competing interest

The authors declare that they have no known competing financial interests or personal relationships that could have appeared to influence the work reported in this paper.
